# Munificence of the King of Prussia for Medical Services

**Published:** 1847-09

**Authors:** 


*Munificence of the King of Prussia for Medical Services.*—The King of Prussia caused to be purchased, a few days ago, the villa of Thiergarton, occupied by Dr. Schoenbein, physician to the Queen, and then sent the doctor the purchase-deeds which bore his name as proprietor, in order to recompense the care and attention which he bestowed on her majesty during her recent illness.—*Med. News.*

